# Assessment of Corneal Biomechanical Properties by CorVis ST in Patients with Dry Eye and in Healthy Subjects

**DOI:** 10.1155/2015/380624

**Published:** 2015-11-08

**Authors:** Qin Long, Jingyi Wang, Xue Yang, Yumei Jin, Fengrong Ai, Ying Li

**Affiliations:** Department of Ophthalmology, Peking Union Medical College Hospital, Chinese Academy of Medical Sciences and Peking Union Medical College, Beijing 100730, China

## Abstract

*Purpose*. To investigate corneal biomechanical properties in patients with dry eye and in healthy subjects using Corneal Visualization Scheimpflug Technology (CorVis ST). *Methods.* Biomechanical parameters were measured using CorVis ST in 28 eyes of 28 patients with dry eye (dry eye group) and 26 normal subjects (control group). The Schirmer I test value, tear film break-up time (TBUT), and corneal staining score (CSS) were recorded for each eye. Biomechanical properties were compared between the two groups and bivariate correlation analysis was used to assess the relationship between biomechanical parameters and dry eye signs. *Results.* Only one of the ten biomechanical parameters was significantly different between the two groups. Patients in the dry eye group had significantly lower highest concavity time (HC-time) (*P* = 0.02) than the control group. Correlation analysis showed a significant negative correlation between HC-time and CSS with marginal *P* value (*ρ* = −0.39, *P* = 0.04) in the dry eye group. *Conclusions.* The corneal biomechanical parameter of HC-time is reduced in dry eyes compared to normal eyes. There was also a very weak but significant negative correlation between HC-time and CSS in the dry eye group, indicating that ocular surface damage can give rise to a more compliant cornea in dry eyes.

## 1. Introduction

Dry eye is a very common condition which is characterized by a lack of tear secretion or excessive tear evaporation that affects tens of millions of people worldwide, with a higher prevalence in Asian population [1, 2]. It is a multifactorial disease of the tears and ocular surface that results in symptoms of discomfort and visual disturbance and signs including tear film instability with potential damage to the ocular surface [3]. Studies show that the cornea is a complex biomechanical composite and that an intact corneal structural component is important for overall corneal biomechanics [4, 5]. Considering the high prevalence of dry eye, its potential influence on corneal biomechanics needs to be clarified.

Many studies have indicated that inflammation plays a key role in the pathogenesis of dry eye related corneal damage [6, 7]. A number of inflammatory cytokines and chemokines have been shown to be consistently elevated in dry eyes [8–10] and may have potential impact on corneal tissue and consequently alter the corneal biomechanical behavior. Some evidence has shown significant corneal biomechanical alterations in eyes with glaucoma and keratoconus [11, 12], which are also considered to have ocular surface inflammation [13, 14] indicating a possible similar mechanism of action in dry eye.

Since dry eye is initially a disorder of the tear film layer, tear secretion value, tear film break-up time (TBUT), and corneal staining score (CSS) are valuable parameters to assess the severity of dry eye and ocular surface integrity. As we mentioned that an intact corneal structural component is essential to maintain the corneal biomechanics, therefore, the above parameters can be applied to evaluate the impact of dry eye on the behavior of corneal biomechanics.

Age is also a potential factor for the corneal biomechanical alterations in dry eyes since it has been reported that the prevalence of dry eye increases significantly with age [2]. Until now, no reports have addressed the relationship between age and corneal biomechanics in dry eyes.

The Ocular Response Analyzer (ORA) (Reichert, Buffalo, NY, USA) was the first commercially available device to measure the in vivo corneal biomechanical properties of corneal hysteresis (CH) and corneal resistance factor (CRF) [15, 16]. In a study using the ORA, Firat and Doganay reported that corneal biomechanical parameters such as CH and CRF were not influenced in dry eye [17].

Corneal Visualization Scheimpflug Technology (CorVis ST) (Oculus Optikgeräte GmbH, Wetzlar, Germany) is a recently developed noncontact tonometry system since 2011. With an integrated ultra-high-speed Scheimpflug Camera, it is able to record real-time dynamic deformation of the cornea, allowing direct description of the corneal biomechanics for clinical evaluation [18]. Until now, biomechanical parameters generated from CorVis ST have been recorded for glaucoma and diabetes mellitus and after refractive procedures [19–22].

Herein, the aims of this study are twofold: (1) to compare the corneal biomechanical parameters of patients with dry eye and normal subjects by the CorVis ST and (2) to assess the correlation between corneal biomechanical parameters and other characteristics, such as age and dry eye parameters.

## 2. Methods

In this observational comparative study, unrelated Chinese patients with or without dry eye were recruited sequentially from the Department of Ophthalmology, Peking Union Medical College Hospital, Beijing, China. The study was performed according to the Declaration of Helsinki. Informed consent was obtained from all subjects prior to participation in the study.

The inclusion criteria of dry eye were identified according to the consensus of dry eye disease in China (2013): (1) at least 1 of 6 symptoms: dryness, gritty/sandiness, burning, tiredness, discomfort, and blurred vision with TBUT (the time to initial break-up of the tear film following a blink) for less than 5 seconds (s) using FLUOR-STRIP (Tianjin Jingming New Technological Development Co., Ltd., Tianjin, China) or a Schirmer I test (without anesthesia, eye closed during the test) value of less than 5 mm per 5 minutes using SCHIRMER TEAR TEST STRIPS (Tianjin Jingming New Technological Development Co., Ltd., Tianjin, China); (2) at least 1 of 6 symptoms: dryness, gritty/sandiness, burning, tiredness, discomfort, and blurred vision with 5 s < TBUT ⩽ 10 s or 5 mm/5 min < Schirmer I test (without anesthesia) ⩽ 10 mm/5 min, accompanied by CSS (the score evaluated by employing fluorescein) total of +1 or more [scale 0 (none) to 12 (severe)], as described in Table 1. TBUT and CSS were observed using slit lamp biomicroscopy by the same masked investigator; Schirmer I test was performed more than 20 minutes after dye staining by another masked investigator.

Patients were excluded from the study if they had concurrent ocular infectious disease, ocular inflammatory disease other than dry eye, a positive history of ocular surgery, ocular or systemic diseases (e.g., corneal scars, corneal dystrophy, corneal degradation, keratoconus, glaucoma, uveitis, systemic autoimmune diseases, and diabetes mellitus), or local or systemic medication use other than artificial tears. In addition, subjects with a refractive error greater than ±1.00 D or contact lens wearers were excluded from the study.

**Figure 1:**
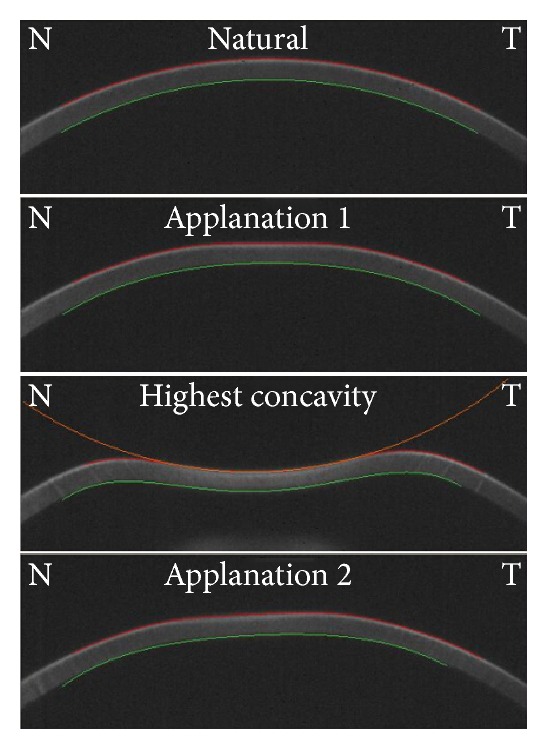
The corneal deformation during air puff from CorVis ST. Due to the air puff, the cornea starts with a natural convex shape and undergoes three distinct phases of first applanation, highest concavity, and second applanation, respectively.

Corneal biomechanical parameters were obtained using CorVis ST (Type 72100, Oculus Optikgeräte GmbH, Wetzlar, Germany) by one masked investigator in every case to eliminate any possible interobserver variability more than 20 minutes after Schirmer I test. CorVis ST uses a high speed Scheimpflug camera (4330 frames/s), covering 8.0 mm horizontally, and records 140 images of the corneal deformation in response to a puff of air. Due to the air puff, the cornea underwent three distinct phases: first applanation, the highest concavity, and second applanation, respectively (Figure 1). Ten phase-specific parameters were automatically generated during the process (Table 2) [23]. Intraocular pressure (IOP) and central corneal thickness (CCT) were also obtained during one measurement procedure. Only the acquisitions showing “OK” on quality of scan (QS) were analyzed.

To reduce the potential diurnal variability, all the measurements were performed between 8:00 and 11:00 a.m.

## 3. Data Analysis

Data were analyzed using IBM SPSS 19.0 for Windows statistical software (SPSS, Chicago, IL) and GraphPad Prism 5 (GraphPad Software, Inc.). Numerical variables were presented as mean ± SD. Shaphiro-Wilk test was used to test normal distribution. Two-tailed Student's *t*-test and Mann-Whitney *U* test were used to compare the observational parameters of the two groups depending on data normality. Pearson or Spearman bivariate correlation analysis was used according to data normality to assess the relationship between corneal biomechanical parameters and potential related characteristics, such as age, IOP, CCT, and dry eye parameters. The level of statistical significance was set to *P* < 0.05. Due to the significant correlation between the right and left eyes, only one randomly selected eye from each subject was included in the analysis.

## 4. Results

Overall, 54 patients were included in the study. The dry eye group (*n* = 28) included 18 female and 10 male patients, with a mean age of 46.82 years (range, 14 to 68 years; mean ± SD, 46.82 ± 14.42 years). 20 female and 6 male patients constituted the control group, with a mean age of 40.19 years (range, 24 to 67 years; mean ± SD, 40.19 ± 11.39 years). There were no differences between the two groups in terms of age (*t* = 1.50, *P* = 0.15) and sex (*X* ^2^ = 1.03, *P* = 0.31). Significant differences were found between the dry eye group and control group in terms of Schirmer I test value (mean ± SD, 2.43 ± 1.85 mm versus 12.65 ± 5.92 mm; Mann-Whitney *U* = 2.5, *P* < 0.001), TBUT (mean ± SD, 3.07 ± 1.76 s versus 7.19 ± 2.38 s; Mann-Whitney *U* = 55.5, *P* < 0.001), and CSS (mean ± SD, 1.11 ± 1.83 versus 0.04 ± 0.20; Mann-Whitney *U* = 243.5, *P* = 0.003).

**Figure 2:**
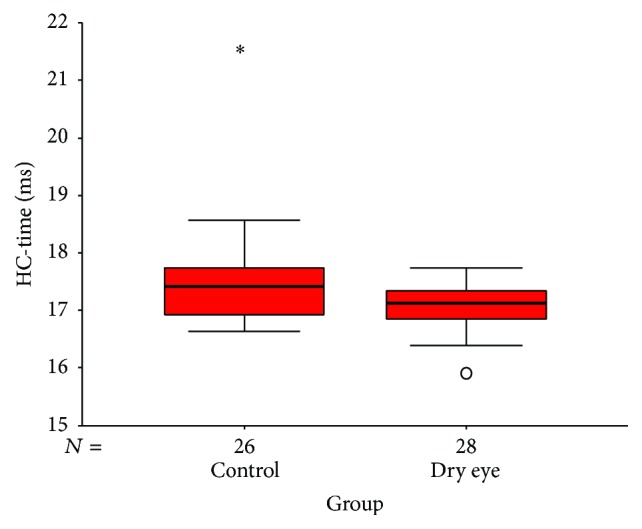
Box plot shows the distribution percentage difference between the dry eye group and control group for HC time (time from starting until the highest concavity is reached). The median for each data set is indicated by the center line, and the first and third quartiles are represented by the edges of the area, which is known as the interquartile range (IQR). The 95%/5% confidence intervals are represented by the ends of the lines extending from the IQR. The circles denote the outliers with values of more than 1.5 IQR from the upper or lower edge of the box.

The corneal biomechanical parameters and IOP and CCT values are shown in Table 3. The differences in IOP and CCT were not statistically significant between the dry eye group and control group (IOP: *t* = 0.15, *P* = 0.88; CCT: *t* = 0.13, *P* = 0.90). Only one of ten biomechanical parameters was significantly different between the dry eye group and control group. Patients in the dry eye group had a significantly lower time at highest concavity (HC-time) (Mann-Whitney *U* = 223.0, *P* = 0.02) compared to the control group (Figure 2).

**Figure 3:**
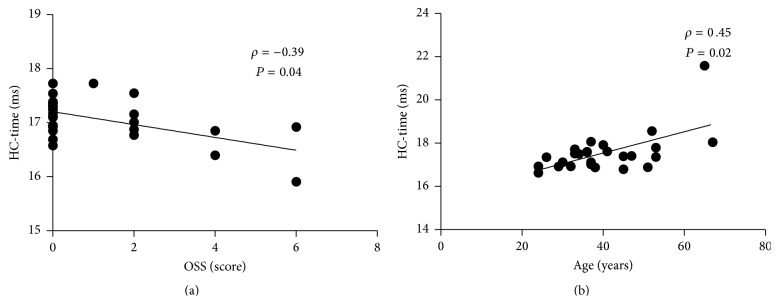
Scatter diagrams of bivariate correlation analysis. (a) Correlation between the HC- time (time from starting until the highest concavity is reached) and CSS (corneal staining score). (b) Correlation between the HC-time and age; *ρ*: Spearman's correlation coefficient value.

In the dry eye group, bivariate correlation analysis showed a significant negative correlation between HC-time and CSS with marginal *P* value (Spearman *ρ* = −0.39, *P* = 0.04) (Figure 3(a)). No correlation was found between HC-time and age, sex, Schirmer I test value, and TBUT (Spearman correlation analysis, all *P* > 0.05). In contrast, bivariate correlation analysis of the control group showed a significant positive correlation between HC-time and age (Spearman *ρ* = 0.45, *P* = 0.02) (Figure 3(b)); however, no correlation was noted between HC-time and sex, Schirmer I test value, TBUT, and CSS (Spearman correlation analysis, all *P* > 0.05). There was no correlation between HC-time and IOP and CCT in both groups (Spearman correlation analysis, all *P* > 0.05). The correlation coefficients and *P* values are shown in Table 4.

## 5. Discussion

The integrity of the cornea is dependent on its biomechanical properties of elasticity, viscosity, and viscoelasticity, which in turn can be affected by the integrity of epithelial barrier, collagen fibrils arrangement, regional pachymetry, hydration, and age [24]. Knowledge of the contribution of corneal biomechanics to dry eye is essential to develop appropriate treatment strategies particularly in cases with concurrent conditions, such as glaucoma and keratoconus, as well as predicting the response to clinical procedures, such as corneal transplant, refractive surgery, and corneal collagen cross-linking [24, 25]. To date, there are 2 systems available for clinical use, ORA, and the recently developed CorVis ST, which are able to provide dynamic quantitative information, to precisely evaluate corneal biomechanics. To our knowledge, this is the first study to use CorVis ST to investigate corneal biomechanics in dry eyes.

This study demonstrated that patients in the dry eye group had significantly lower HC-time than age- and sex-matched normal controls. Bivariate correlation analysis showed a significantly negative correlation between HC-time and CSS in the dry eye group and a significantly positive correlation between HC-time and age was noted in control group, but not in the age- and sex-matched dry eye group.

HC-time represents the time from commencement until the highest concavity is reached and reflects the time to maximum deformation. A shorter HC-time may be due to a more compliant cornea reaching the highest concavity. Studies have indicated that HC-time was significantly shorter after laser in situ keratomileusis (LASIK) compared to small incision lenticule extraction (SMILE). Since the major difference between the two refractive procedures is the flap, the lower HC-time after LASIK may result from more collagen fibres being cut during flap creation [26]. For dry eyes, there is a consensus that dry eye is an inflammation triggered disease, since significantly increased inflammation factors and proteins have been detected in the tear film and ocular surface tissue of dry eye [9, 11, 27], which may have potential impact on corneal biomechanics. For example, matrix metalloproteinase 9 (MMP-9) was shown to be elevated in dry eyes [28, 29]. As a member of matrix metalloproteinases (MMPs) family, MMP-9 is involved in the degradation of extracellular matrix components (ECM) and contributes to inflammation, wound healing, and tissue remodeling [30, 31]. It is thought that the increased MMP-9 expression may cause a more compliant cornea in dry eye, and this hypothesis is supported by our study. We found a lower HC-time in dry eye patients compared to age- and sex-matched normal controls. However, none of the remaining CorVis ST parameters supported this theory. Given that corneal biomechanical behavior is governed by the stroma, therefore, greater alterations would be expected in severe dry eyes which exhibit greater corneal stromal lesions.

Corneal biomechanical properties can be affected by multiple factors, including the integrity of epithelial barrier [5]. Elsheikh et al. showed that an intact corneal epithelium has a very important function over the corneal biomechanics [5]. The integrity of the corneal epithelium can be represented by CSS with fluorescein staining [3]. The negative correlation between HC-time and CSS in dry eye group suggests that greater corneal epithelium damage results in more compliant cornea, resulting in a shorter time to reach the highest concavity. Since only a borderline *P* value was noted, further studies should be conducted to confirm this significance.

The evaluation of CCT and corneal epithelial damage are essential to study and compare the biomechanical parameters between the dry eye group and control group. In our study, we did not find difference in terms of CCT between two groups; this was consistent with the study by Firat and Doganay [17]. However, CCT was observed to be thinner in dry eyes compared to normal controls by Meyer et al. [32]. With regard to the evaluation of corneal epithelial damage, the measurements of central corneal epithelial thickness (subjective assessment) and CSS (objective assessment) are both used in clinic. Similar to CCT, the results of central corneal epithelial thickness were also contradictory. It showed to be thicker in dry eyes in the study of Kanellopoulos and Asimellis [33] and no difference in Cui's study [34]. Further objective assessment approaches, such as epithelial mapping or tear film osmolarity, should be considered to clarify our results in the future.

Age related alterations in corneal biomechanics are associated with corneal stiffening and decreasing viscoelasticity [35]. Ex vivo and in vivo studies have demonstrated that corneal stiffness changes with age, accompanied with stromal microstructure changes including the more cross-links of collagen fibrils within the cornea [36, 37]. Several studies showed that CH and CRF significantly decreased with age in healthy population [38, 39]. Other authors, however, demonstrated no correlation between corneal biomechanics and age by ORA [40]. With CorVis ST, Valbon et al. reported that only the HC-time correlated significantly with age in healthy eyes [23]. A similar result was seen in our study, and data suggested that older subjects tended to have longer HC-time due to a less compliant cornea. However, we did not find the significant correlation between HC-time and age in dry eye group, which may be due to the neutralization of the impact caused by aging and the integrity of corneal epithelium because of dry eye.

The main limitations of our study are that (1) the sample size is relatively small; (2) not all the subjects had a general examination and we excluded systemic diseases only by the history; (3) we did not test the diurnal variation for the corneal biomechanics, although several studies reported a stable profile during daytime acquisitions by ORA [41] (to date, no study has addressed diurnal variation by CorVis ST; therefore, the importance of this factor in our study is unknown); and (4) biomechanical properties can be affected by corneal hydration, but, in dry eye patients, it is difficult to eliminate the influence caused by the use of artificial tears.

In summary, the present study showed that HC-time was significantly lower in dry eye patients than in age- and sex-matched normal controls. Correlation analysis showed a very weak but significant negative correlation between HC-time and CSS in the dry eye group, indicating that the ocular surface damage can cause a more compliant cornea in dry eyes. As CorVis ST is a relatively new technology, further studies with a larger sample size should be performed to elucidate its full usefulness for dry eye patients, as this might be helpful in clinical practice, especially for planning ophthalmological interventions, such as refractive surgery, which has been reported to alter corneal biomechanical behavior due to corneal tissue removal.

## Figures and Tables

**Table 1:** Grading of cornea staining.

Score	Cornea staining (with fluorescein)
0	0 dots
1	1–30 dots
2	>30 dots without confluence
3	>30 dots with confluence, filament, or ulcer

Staining is represented by punctate dots on the cornea, the cornea is divided into four quadrants, and the total cornea staining score is represented by the total score of four cornea quadrants.

**Table 2:** All biomechanical parameters derived from CorVis ST.

CorVis ST parameters	Means
A1-time	Time from starting until the first applanation
A2-time	Time from starting until the second applanation
A1-length	Cord length of the first applanation
A2-length	Cord length of the second applanation
A1-velocity (A1-V)	Corneal speed during the first applanation moment
A2-velocity (A2-V)	Corneal speed during the second applanation moment
Highest concavity-time (HC-time)	Time from starting until HC is reached
Peak distance (PD)	Distance between the two peaks of the cornea at HC
HC radius	Central concave curvature at HC
Deformation amplitude (DA)	Maximum amplitude at the highest concavity

**Table 3:** All parameters derived from CorVis ST in dry eye group and control group, mean ± SD.

Parameters	Dry eye group (*n* = 28)	Control group (*n* = 26)	*t*/*U* value	*P* value
A1-time (ms)	7.38 ± 0.28	7.37 ± 0.16	0.05^*∗*^	0.96
A2-time (ms)	21.85 ± 0.42	21.95 ± 0.29	0.91^*∗*^	0.37
A1-length (mm)	1.75 ± 0.05	1.77 ± 0.03	309.0^#^	0.34
A2-length (mm)	1.71 ± 0.23	1.77 ± 0.19	301.5^#^	0.28
A1-V (m/s)	0.15 ± 0.02	0.15 ± 0.01	0.22^*∗*^	0.83
A2-V (m/s)	−0.30 ± 0.07	−0.31 ± 0.05	0.23^*∗*^	0.82
HC-time (ms)	17.07 ± 0.40	17.55 ± 0.95	223.0^#^	0.02
PD (mm)	3.92 ± 1.19	3.89 ± 1.16	353.0^#^	0.86
HC radius (mm)	7.29 ± 0.90	7.15 ± 0.95	317.0^#^	0.42
DA (mm)	3.92 ± 1.19	3.89 ± 1.16	0.42^*∗*^	0.68
IOP (mmHg)	13.64 ± 2.76	13.70 ± 1.61	0.15^*∗*^	0.88
CCT (*μ*m)	534.82 ± 25.64	537.0 ± 35.97	0.13^*∗*^	0.90

A1-V: A1-velocity; A2-V: A2-velocity; HC-time: highest concavity-time; PD: peak distance; HC radius: radius at HC; DA: deformation amplitude; IOP: intraocular pressure; CCT: central corneal thickness; ^*∗*^ *t*-test value; ^#^Mann-Whitney *U* test value.

**Table 4:** Factors associated with HC-time.

Parameters	Dry eye group (*n* = 28)		Control group (*n* = 26)	
	*ρ* value	*P* value	*ρ* value	*P* value
Age	−0.04	0.82	0.45	0.02
Gender	0.05	0.80	0.33	0.10
Schirmer I test value (mm)	−0.16	0.41	−0.35	0.08
TBUT (s)	0.02	0.90	−0.22	0.28
CSS (score)	−0.39	0.04	0.04	0.85
IOP (mmHg)	0.17	0.38	−0.28	0.17
CCT (*μ*m)	0.06	0.76	0.15	0.46

TBUT: tear break-up time; CSS: corneal staining score; IOP: intraocular pressure; CCT: central corneal thickness; Coeff: the correlation coefficient; *ρ*: Spearman's correlation coefficient value.
